# Putting episodic disability into context: a qualitative study exploring factors that influence disability experienced by adults living with HIV/AIDS

**DOI:** 10.1186/1758-2652-12-30

**Published:** 2009-11-09

**Authors:** Kelly K O'Brien, Aileen M Davis, Carol Strike, Nancy L Young, Ahmed M Bayoumi

**Affiliations:** 1 Department of Health Policy, Management and Evaluation, Faculty of Medicine, University of Toronto, Ontario, Canada; 2 Centre for Research on Inner City Health, The Keenan Research Centre in the Li Ka Shing Knowledge Institute, St Michael's Hospital, Toronto, Ontario, Canada; 3 Division of Health Care and Outcomes Research and Arthritis and Community Research and Evaluation Unit, Toronto Western Research Institute, Toronto, Ontario, Canada; 4 Department of Psychiatry, University of Toronto, Toronto, Ontario, Canada; 5 Centre for Addiction and Mental Health, Toronto, Ontario, Canada; 6 School of Rural and Northern Health, Laurentian University, Sudbury, Ontario, Canada; 7 Faculty of Health Sciences, School of Rehabilitation Science, McMaster University, 1400 Main Street West, Room 403, Hamilton, Ontario, Canada

## Abstract

**Background:**

An increasing number of individuals may be living with the health-related consequences of HIV and its associated treatments, a concept we term disability. However, the context in which disability is experienced from the HIV perspective is not well understood. The purpose of this paper is to describe the contextual factors that influence the experiences of disability from the perspective of adults living with HIV.

**Methods:**

We conducted four focus groups and 15 face-to-face interviews with 38 men and women living with HIV. We asked participants to describe their health-related challenges, the physical, social and psychological areas of their life affected, and the impact of these challenges on their overall health. We also conducted two validity check focus groups with seven returning participants. We analyzed data using grounded theory techniques to develop a conceptual framework of disability for adults living with HIV, called the Episodic Disability Framework.

**Results:**

Contextual factors that influenced disability were integral to participants' experiences and emerged as a key component of the framework. Extrinsic contextual factors included social support (support from friends, family, partners, pets and community, support from health care services and personnel, and programme and policy support) and stigma. Intrinsic contextual factors included living strategies (seeking social interaction with others, maintaining a sense of control over life and the illness, "blocking HIV out of the mind", and adopting attitudes and beliefs to help manage living with HIV) and personal attributes (gender and aging). These factors may exacerbate or alleviate dimensions of HIV disability.

**Conclusion:**

This framework is the first to consider the contextual factors that influence experiences of disability from the perspective of adults living with HIV. Extrinsic factors (level of social support and stigma) and intrinsic factors (living strategies and personal attributes) may exacerbate or alleviate episodes of HIV-related disability. These factors offer a broader understanding of the disability experience and may suggest ways to prevent or reduce disability for adults living with HIV.

## Background

Since the advent of combination antiretroviral therapy, an increasing number of individuals may be living with disability [1,2], herein defined as the health-related consequences experienced as a result of HIV and its treatments. To fully understand the disability experience, one should consider the context in which these HIV health-related consequences occur, such as a person's physical, social or political environment and his or her personal characteristics, as these may represent possible targets for health interventions or policy changes to prevent or reduce disability. For this study, we defined contextual factors as features that interact with and influence disability experienced by adults living with HIV.

A framework in which to understand the disability context specifically from the perspective of people living with HIV has not previously been developed. Early generic disablement frameworks, such as the Nagi Scheme and International Classification of Impairments, Disabilities and Handicaps (ICIDH), provided insight into factors that shaped the disability experience, but did not explore the detailed role of contextual factors [3-5]. More recent frameworks, such as the Handicap Creation Process, Disablement Process and International Classification of Functioning, Disability and Health (ICF), acknowledged the individual and environmental factors that influence disability [6-8].

However, we were uncertain whether these frameworks and their contextual factors fully captured the complex disability experience specific to HIV. Understanding the HIV disability experience in the form of a framework is important for adults living with HIV, health providers and policy makers to be able to adequately measure and address the disablement needs of this population.

We developed the Episodic Disability Framework based on the experiences of adults living with HIV [9]. This framework conceptualizes disability as multi-dimensional and episodic in nature, characterized by unpredictable periods of wellness and illness. Disability spans physical, mental, emotional and social life domains. Episodes of disability are described as health-related setbacks that manifest from HIV disease, its treatments or conditions.

**Figure 1:**
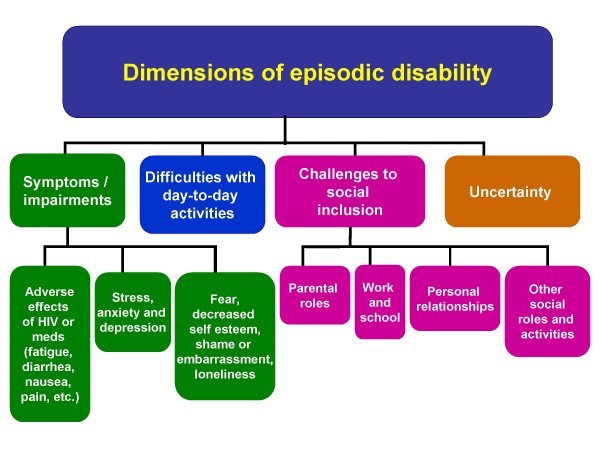
**Dimensions of episodic disability**. Four dimensions of *episodic disability *and their sub-components that may be experienced by adults living with HIV.

The framework consists of three components: 1) dimensions, 2) contextual factors, and 3) triggers of disability. Dimensions of episodic disability include symptoms and/or impairments, difficulties carrying out day-to-day activities, challenges to social inclusion, and uncertainty that may fluctuate on a daily basis and over the entire course of HIV (Figure 1). Triggers of major or momentous episodes of disability include such life events as receiving an HIV diagnosis, starting or changing antiretroviral medications, experiencing a serious illness, and suffering a loss of others. These components are discussed in detail elsewhere [9].

The purpose of this paper is to describe the second component of this framework, contextual factors that influence disability, which emerged as an integral feature of participants' disability experience.

## Methods

We used qualitative methods of inquiry and analysis to seek understanding of the contextual factors that influence disability from the perspective of adults living with HIV [10,11]. We recruited adults living with HIV (18 years and over) who self-identified as having experienced at least one health-related consequence attributed to their HIV illness from a hospital, primary care clinic or AIDS service organization in downtown Toronto, Ontario, Canada. We used theoretical sampling whereby we recruited participants based on emerging themes related to disability [10]. A Community Advisory Committee, comprised of people living with HIV, representatives from AIDS service organizations and the Ontario Ministry of Health and Long Term Care, guided all aspects of this research. St Michael's Hospital and the University of Toronto Research Ethics Boards approved the study protocol.

This research consisted of three phases of inquiry: pilot focus groups, semi-structured face-to-face interviews, and validity check focus groups. We asked participants to characterize their day-to-day health-related challenges living with HIV, and to describe how such challenges affected their physical, social and psychological wellbeing and their overall health. The goal of the pilot focus groups was to guide the sampling strategy and refine the interview guide for the interview phase of the research; these data also contributed to the development of the conceptual framework [12]. After analyzing the pilot focus group transcripts, we conducted face-to-face interviews with new participants to develop the framework, including contextual factors that influence the disability experience. After analysis of the interview data, all interview participants were invited to participate in a final validity check focus group to share preliminary findings and discuss the extent to which the draft framework and its contextual factors represented their experiences. Participants were asked to comment on the contextual factors, their terminology and how they influenced their disability experience.

Interviews and focus group discussions were conducted at three community-based organizations. Field notes were taken throughout, and all discussions were audio-taped and later transcribed verbatim for analysis. Data management was facilitated using N6 Qualitative Software [QSR International Pty. Ltd; 2002]. All participants completed a demographic questionnaire and the HIV Symptom Index [13].

### Analysis

We used grounded theory techniques to analyze combined pilot focus group and interview data to develop a framework of disability [14]. We used a systematic set of procedures, which included: coding transcriptions line by line into concepts termed meaning units; grouping similar meaning units into categories; comparing categories with other categories; identifying relationships between categories; and integrating these categories to develop the disability framework [14].

We examined the validity check focus group data for ways to refine the preliminary framework for content, terminology and interrelationships, and to determine whether the identified contextual factors adequately represented participants' experiences.

We used theoretical saturation, constant comparative analysis and validity checks to enhance rigour [15]. Theoretical saturation meant that we recruited participants and collected data until no "new" relevant data emerged. We used a constant comparative analysis whereby data collection and analysis occurred simultaneously, ensuring discussion guides continually evolved so that questions adequately built and strengthened the contextual factors.

Validity checking with the final focus groups involved sharing preliminary results with the participants, and enabling them to confirm, refute or enhance the contextual factors within the framework. Meaning units and categories of three transcripts were also independently assigned by three authors and a colleague with expertise in qualitative research. Authors and the Community Advisory Committee formally reviewed interim data and analytical interpretations six times over the course of 14 months.

## Results

Thirty-eight participants took part in one of four pilot focus groups (23 participants) or one of 15 face-to-face interviews (15 participants). Of the pilot focus groups, two included men only, one women only, and the other both men and women. Seven of the interview participants (three men, four women) returned for one of two validity check focus groups. The first validity check focus group included men and women (five participants) and the second included women only (two participants). Characteristics of participants varied in relation to gender, age, ethnocultural background, length of time since HIV diagnosis and antiretroviral use (Table 1).

**Table 1:** Participant characteristics (n = 38)

Characteristic	Number (%)
*Gender*	
Male	21 (55%)
Female	16 (42%)
Transgendered	1 (3%)

Mean age (years) (range)	41 (27-58)

Identified with a particular ethnic group	23 (60%)**

Nadir CD4 count <200 cells/mm^3^	19 (50%)

Diagnosed prior to 1996	17 (45%)

Experienced an HIV-related illness	11 (73%)*†

Currently taking HIV medications	25 (66%)

Currently working	6 (40%)* (3 full time, 3 part time)

*Self-rated health status*	
Poor	0 (0%)
Fair	2 (5%)
Good	16 (42%)
Very good	15 (39%)
Excellent	5 (14%)

*HIV Symptom Index*	
Median number of symptoms present	15/20 (IQR: 8-18)
Median number of bothersome symptoms	13/20 (IQR: 8-18)^

*denominator of 15 interview participants only

**13 identified themselves as African/African Caribbean/black African/black; 2 Jewish; 1 West Indian; 1 Latin; 1 Italian Canadian; 1 Irish Canadian; 1 French Italian; 1 English British; 1 white caucasian; 1 not specified.

†most common HIV-related illness included pneumonia/lung infection/PCP pneumonia (n = 6 participants).

^ Most bothersome symptoms included fatigue or loss of energy, feeling sad, down or depressed, and feeling nervous or anxious.

IQR - interquartile range

### Contextual factors of disability

**Figure 2:**
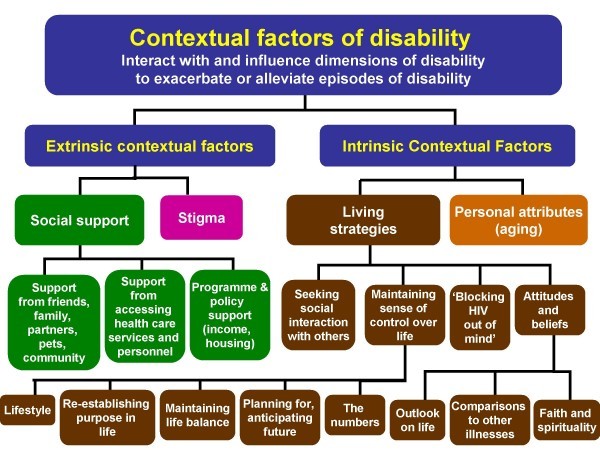
**Contextual factors of disability**. Factors that describe the context in which disability is experienced. Extrinsic and intrinsic contextual factors could exacerbate or alleviate each of the four dimensions of disability for adults living with HIV.

Factors that altered disability were integral to participants' experiences, and emerged as a key component of the Episodic Disability Framework. From these data, we identified four factors that participants described as influencing their disability: social support, stigma, living strategies and personal attributes. We classified these contextual factors as either intrinsic or extrinsic (Figure 2). Extrinsic factors originated from the external environment, whereas intrinsic factors represented attributes internal to the individual.

Contextual factors could be static (either present or absent), progressive or fluctuate over time. Based on the nature and level of severity, these contextual factors were perceived by participants to influence the episodic nature of disability. We describe each factor and analyze how they influence the dimensions of disability within the Episodic Disability Framework.

### Extrinsic factors

#### Social support

Social support was perceived to alleviate disability, whereas a lack of support exacerbated disability and included three main sources: support from friends and family; support from the health care services and personnel; and programme and policy support (Figure 2).

Practical and emotional support from friends, family, partners, pets and the community eased episodes of disability:

Right now I'm working on my mental state. I have wonderful friends who are helping me and a wonderful partner now who is trying to keep me inspired ... (Focus Group (FG)-3; Participant 2; man in his 30s)

In contrast, a lack or absence of support exacerbated symptoms and/or impairments, such as feelings of loneliness or depression:

I'm alone a lot. Unfortunately, I don't have that many friends. I don't have that many people to drop in and visit me. (Interview (INT)-7; man in his 40s)

The ability to access health care services and personnel was perceived by participants to reduce episodes of disability. Participants recounted how practical support from personal support workers helped them successfully complete their day-to-day activities, such as cleaning, cooking and doing laundry. Others described emotional support from physicians and other health providers who gave them "light at the end of the tunnel" (INT-3). Many felt confident with their physicians' knowledge and skills, which offered a sense of comfort and assurance, reducing their uncertainty of living with HIV:

I am privileged. I have amazing doctors ... They're really compassionate, their bed-side manner, and they're really bright, so they're always on top. And I know they're not going to just let something bad happen to me, they're always on top of my blood work, and looking for the smallest glitch... that's my sense of safety... (INT-10; woman in her 20s)

In contrast, participants who perceived that they lacked health care support struggled with day-to-day activities and reported increased stress, anxiety and depression living with HIV:

It's a challenge, having to rely on yourself most of the time, 'cause you just can't get the support that you need. (INT-7; man in his 40s)

Participants received programme and policy support from a range of public and private insurance and benefit programmes, including the Ontario Disability Support Program, Canada Pension Plan, private insurance plans, and housing and food bank services. While some participants reported having adequate support (e.g., workplace health insurance plans), others struggled to live on a limited and sometimes sporadic income. Low levels of income support reduced participants' ability to engage in financially dependent social activities (e.g., going to the movies with friends) and exacerbated challenges to social inclusion. Participants who lacked supportive workplace health insurance plans described living with heightened uncertainty, worrying what might happen if they had to leave work due to an illness.

#### Stigma

Stigma emerged as an extrinsic factor that exacerbated participants' experiences of disability (Figure 2). Stigma was experienced from family, work colleagues, employers and health care providers due to participants' HIV status, sexual orientation, ethnocultural background, employment status and/or gender. Stigma commonly exacerbated symptoms and/or impairments (e.g., decreased self-esteem, increased stress, anxiety and depression, and shame and embarrassment) and challenges to social inclusion (e.g., inability to work, and difficulty initiating or maintaining personal relationships):

I feel when I'm around certain people or certain ethnicities, I have to hide who I am ... HIV shouldn't define us as people ... We are who we are, but because of the stigma that comes with it ... that's what I've been struggling with. It's being able to overcome that stigma that lies within me, because a woman, a woman with no job, and then being infected ... (FG-4; Participant 7; woman in her 30s)

Some participants who were living with HIV for many years felt stigmatized by family members, which exacerbated levels of depression:

Some of the family hasn't accepted it... that will bring me a little depressed... (INT-13; man in his 30s)

Some felt stigmatized by beliefs of others who felt they deserved becoming infected "...because of the behaviour that led to it" (INT-4), which reduced self-esteem. Some participants chose not seek new personal relationships due to stigma experienced after disclosing their HIV status:

I haven't been dating in a long time because of this. Meeting people and then explaining to them ... and saying, "Oh, I have HIV and blah blah blah." And then I discover awfully, I was so disillusioned, I find out that they knew nothing. And then they would be all freaked out ... (INT-10; woman in her 20s)

Others successfully "learned to live with it" (INT-6) by avoiding negative consequences of stigma:

People make it a big issue, I don't. I don't live and dwell on it day to day. I mean it is still hard, because certain people don't accept it ever, but I don't make it an issue for myself or for my kids. We don't live that every day, breathe that every day... (INT-9; woman in her 30s)

### Intrinsic factors

#### Living strategies

Living strategies was a term designated by participants, which included behaviours, attitudes and beliefs that participants adopted in order to deal with HIV and its resulting disability, including: seeking social interaction with others; maintaining a sense of control over life and the illness; "blocking HIV out of the mind"; and adopting attitudes and beliefs to help manage living with HIV (Figure 2).

Some participants sought social interaction through new personal and professional relationships to enhance their social inclusion:

The big thing ... is getting involved in life. And life means people ... they could be my co-workers, they could be anybody. Just to be involved, make friends, have a life! (INT-3; man in his 40s)

In contrast, others purposefully isolated themselves or avoided others, particularly during periods of illness in order to minimize burdening loved ones:

I tend to withdraw a little bit until I'm feeling better, and then go back into the world. This isn't necessarily a good thing, though. So when I do have those bad periods, I do withdraw from society. (INT-15; man in his 30s)

For some who had lost loved ones to HIV, they chose to "build a wall" and " [tried] not to get too close to people" (INT-6) to avoid exposing others to similar types of loss if they were to become ill. Isolating oneself amplified social exclusion by reducing opportunities to initiate or maintain personal relationships and exacerbated symptoms and/or impairments, specifically feelings of loneliness and depression. Others viewed self-imposed isolation as a way of reducing stress by protecting friends and family from difficult periods of illness.

Many participants felt that their sense of control over their lives influenced their disability. Participants described how re-evaluation of their lifestyle could enable self control to maximize their health, such as getting adequate nutrition and sleep, reducing substance use, adhering to medications, exercising, avoiding external stressors and ensuring financial security:

As long as I get my rest and eat properly ... and take my medications, to 100% adherence, which I do, then life is livable. (INT-4; man in his 40s)

A second strategy to gain control was to re-establish a sense of purpose as a person living with HIV. Many described having to learn to live with HIV by redefining their baseline level of health in the HIV context. Accordingly, new life goals were adopted, which for some, focused on maximizing their health:

Right now my life is just survival, basically ... not putting my life in danger, not being self-destructive. That takes a lot of my focus ... day- to-day survival, mentally and physically ... look after the basics, like diet and exercise, and rest. (INT-4; man in his 40s)

Others focused more on employment goals to avoid feeling identified by their HIV status:

Sometimes it may benefit a lot of PHAs to go back to work because ... they have a purpose in life. And they can do something other than focus on themselves and their sadness and their depression, and their HIV. (INT-11; woman in her 30s)

A third control strategy related to achieving a healthy life balance. Establishing a daily structure or routine enabled participants to "prioritize" and "not overdo it" (INT-13). Prioritizing allowed participants to plan for a potential episode of disability. Making "to-do" lists prevented participants from feeling overwhelmed by medical appointments and antiretroviral regimens, while achieving a sense of accomplishment after completing the tasks set forth:

I set limitations ... I always have lists ... this is what I'm going to get done today ... Cause then you check it off and wow, I've done this. I set a realistic list and an unrealistic list, so if I get some of the unrealistic done, it's a bonus... Every day there are interruptions that you don't expect ... (INT-12; woman in her 30s)

In contrast, other participants described a lack of balance, which exacerbated episodes of disability. Despite the known consequences to their health, participants "over [did] it" in some situations, working long hours or cleaning their households incessantly, stating it gave them a "burst of energy", a sense of accomplishment, and a reminder that they still have a life to lead:

I don't have a balance ... I have no boundary ... I'm the kind of person that ... will push and push, and just keep doing things until I just crash. I don't know how I do all my kids' laundry, I clean my house, I clean the backyard, I go do groceries, everything, until I just crash ... I think maybe that's what makes me feel better. So I do it, until I get sick I want to tell myself I'm alive. I can still do things, but at the end of the day, I crash. (INT-9; woman in her 30s)

A fourth strategy for maintaining control was planning for and anticipating the future. Some participants transitioned from "living for the present" (INT-15) while preparing for imminent death, to having to plan for their future living with a chronic illness. Because participants could not always prevent nor anticipate episodes of disability, they used planning and preparation to reduce uncertainty:

I work as much as I can when I'm healthy. So I can actually prepare for the future in case I'm sick I'm extremely determined, more than I've ever been, to build something so that in case I get sick. It's always on my mind. (INT-12; woman in her 30s)

A final strategy for maintaining control was paying attention to "the numbers", including CD4 count and viral load. For some participants, these represented "what it means to be healthy" (INT-14). Some considered "the numbers" as a way of predicting their survival and longevity, alleviating uncertainty, stress and anxiety associated with HIV:

Like some people watch the stock market. I am totally watching my T cells, and they move, they go up, and I'm like "Yeah" ... I feel so at ease ... I just don't get anxious. And it's easier for me to put things out of my mind ... And I feel confident, I can see myself living 'til I'm 60 - look at all those T cells! And the viral load is down ... So I feel confident, I'm doing really well and I think this indicates that my survival rate is going to be good, I'm going to live longer. Whereas if I had a higher viral load, I'd be like ... I'm going to die. (INT-10; woman in her 20s)

For one participant, "the numbers" influenced his social inclusion, signaling his perceived readiness to re-enter the workforce:

If my T cell count was up ... I'm sure I would feel a lot more ready to bounce back into the fast-paced society. (INT-4; man in his 40s)

Others chose not to pay attention to "the numbers" as they might exacerbate episodes of uncertainty. Instead, they were used only "as a guideline" and not taken "too seriously" (INT-15).

In addition to control, participants spoke of using strategies to "block HIV out of the mind" in order to distance themselves from their HIV and alleviate uncertainty, stress, anxiety and depression. Some described doing anything they could to "push it [HIV] from [their] mind", which commonly involved distracting themselves by focusing on other life activities:

I try to focus on something else ... I shove it out ... sometimes it's just a matter of turning on music really loud and just blocking it out ... I just need to distract myself and get away .... That's how I cope with uncertainty. 'Cause anytime I approach it, it's too overwhelming, and I can't face it. So ... I just drown it out, that's the best thing. (INT-10; woman in her 20s)

While some participants used drugs or alcohol to keep their mind off HIV, others immersed themselves in exercise or work. Participants sometimes perceived this as a destructive strategy, even though it helped alleviate their sense of disability:

I'm depressed momentarily, for a day, or something like that. The good thing is I don't usually linger for a whole day for a bunch of days in depression. And I think a part of that has to do with the fact that now I have things to do, I can't focus on sitting and being depressed ... sometimes I don't know whether I'm just replacing that with a drug or something like that - instead of going out drinking and forgetting about my problems, or forgetting about my depression, I get into my work. (INT-11; woman in her 30s)

Many participants said they actively adopted particular attitudes and beliefs to cope with living with HIV. Participants reported adopting a positive outlook to deal with HIV-related disability. An outlook of hope and optimism alleviated participants from worrying about when their next episode might arise, the severity of that episode and their long term survival living with HIV. They described how they "coped with attitude", knowing episodes will come and go due to the fluctuating nature of the disease and that periods of illness will likely pass over time: "In a couple of weeks, this will be over." (INT-10)

During periods of wellness, participants used a positive outlook to maximally engage in daily and social activities:

It motivates me to do as much as I can while I'm healthy ... I switch the negative to a positive, all of these things can happen to me, I don't know how long I have, I don't know how long I'm going to be well. I'm going to do everything that I can do, that's possible in this day, to motivate myself to accomplish my dreams or my goals or the things I want to do ... (INT-12; woman in her 30s)

In contrast, others struggled to find a sense of optimism, and acknowledged the detrimental influence that despair and a sense of hopelessness had on their disability:

It seems to all be with the attitude. And some people have terrific attitude, and they're so optimistic. And I think they've got half the battle. I've always tended to be a pessimist, and a depressive-anxious sort of type, and that takes a toll on my body. It takes a toll on my life, and my ability to function. (INT-4; man in his 40s)

Comparing personal experiences to others living with HIV or chronic illnesses was another strategy used by participants. Some positioned themselves as "better-off" than others to perceive themselves as "healthy" or "healthier" within the broader illness context:

I think every day is a good day. 'cause I know there are people less fortunate that can't do those things. And they're more in the AIDS category. (INT-4; man in his 40s)

This alleviated stress, anxiety and depression and uncertainty.

Some participants drew on their faith and spiritual beliefs to alleviate HIV-related disability. This included praying to a higher being to increase their sense of strength, hope and optimism living with HIV. Faith and spirituality were used to alleviate uncertainty, depression and fear:

I pray ... I'm a very spiritual person, and I think that kind of helps me. It guides me through all this stuff ... meditating and spirituality if I keep thinking that my glass is half full ... (INT-14; man in his 30s)

#### Personal attributes

Another intrinsic factor perceived by participants as influencing their disability related to their personal attributes, non-modifiable characteristics, such as age and co-morbid illnesses inherent to an individual (Figure 2). For some participants, who had lived with HIV for more than 10 years, the linkage between these characteristics, HIV and disability was unclear. Some indicated that they were uncertain whether their disability was due to HIV, other co-morbid conditions, associated medications, their age, or a combination.

I don't know if that's because of my HIV, because I don't remember before. Or is it because I'm 40 and it's natural?. Even my doctor can't answer my question. (INT-5; woman in her 40s)

Nevertheless, participants suggested that these attributes might exacerbate HIV-related disability, such as symptoms and/or impairments and difficulties carrying out day-to-day activities. Participants expressed concerns that aging and co-morbid conditions introduced uncertainty, particularly among older participants, who had difficulty sorting through the source of their disability and how to best address it.

## Discussion

We identified several contextual factors that exacerbate or alleviate the experience of disability, as described by adults living with HIV. Although previous studies in the field of HIV/AIDS identified the importance of such factors for quality of life and wellbeing, we know of no previous study that has described how such factors influence disability. For example, strong social support is associated with lower levels of depression [16], fewer health-related symptoms [17], and better psychological and physical adaptation to HIV [18]. Stable or enhanced support over time has also been linked with higher CD4 counts [19] and viral suppression for those taking combination antiretroviral therapy [20]. Support networks also may reduce uncertainty by helping individuals seek information, provide support or reassurance, ease discussions about uncertain issues, facilitate coping skills, and provide acceptance or validation. They may even encourage individuals to consider uncertainty as a normal aspect of life, or source of opportunity rather than a negative consequence of HIV [21]. Support in the form of vocational rehabilitation may facilitate social inclusion, such as returning to or remaining in the workforce [22], whereas a perceived lack of social support, including a loss of insurance income benefits or workplace discrimination, may be a barrier to returning to work [23]. Additionally, stigma may exacerbate depression for those living with HIV, another symptom or impairment of disability [24].

Some psychological characteristics of HIV are analogous to elements of the Episodic Disability Framework. For example, "coping" is analogous to the concept of "living strategies", a term preferred by participants. Coping is associated with changes in quality of life [25,26], depression and distress [27,28], and physical, social and role functioning among persons living with HIV [29-31]. Coping strategies are also associated with enhancing social inclusion by fostering positive personal relationships and social support [32,33]. Greater perceived control over illness (internal locus of control) is analogous to "maintaining sense of control". In contrast to a belief that health outcomes are controlled by others, fate or luck (external locus of control), greater control is associated with positive changes in health-related quality of life [34] and less depression and anxiety about death [35]. To attain this control, some participants adjusted their life expectations and viewed their health positively, developing a new meaningful purpose in life [36,37]. A positive outlook on life and regaining a sense of control may also have implications on enhancing social inclusion by facilitating the transition back into the workforce for those who have left the workforce in the past due to their illness [38].

"Outlook on life" and "faith and spirituality" were specific types of living strategies (attitudes and beliefs) in our framework used to alleviate HIV-related disability. Hope and resiliency are integral to living with an unpredictable illness [39], associated with reduced emotional and psychological distress [40,41], and depression [42,43]. Spirituality is linked with reduced depression [44-46], as well as more broadly associated with intrinsic and extrinsic factors, such as effective coping (living) strategies and enhanced social support among people living with HIV [18]. Participants in this and other research used attitudes and beliefs to reduce their uncertainty and stress of living with HIV [47-52]. Some even considered themselves healthier or "better off" since their diagnosis [53-55].

Finally, the impact of personal attributes, such as co-morbid illnesses, gender, and aging, which were most prominently articulated by participants as influencing their disability, is discussed in the HIV literature. Twenty-nine percent of older adults with HIV experience moderate or severe depression due to decreased supports and access to health and social services [56,57]. However, accessing support is more difficult as these individuals are less likely to disclose their HIV status [58,59], placing them at risk of becoming further isolated and depressed [60]. Also, aging may enhance uncertainty in relation to the origin of symptoms or impairments, particularly those that could emerge from multiple sources [61].

### Contextual factors of the Episodic Disability Framework in relation to other frameworks of disability

While we explored disability specific to HIV, contextual factors in the Episodic Disability Framework relate to other existing disablement frameworks and may contribute to the broader understanding of disability. The Nagi Scheme [3,4], and the International Classification of Impairments, Disabilities and Handicaps (ICIDH) [5] do not elaborate on the context in which disablement may be experienced.

The Handicap Creation Process [6,62,63] was the first to consider personal identity and environmental factors that can either be an obstacle or support to an individual's level of function. However, the environmental factors focused solely on handicap at the societal level, neglecting other components of disability. In the Disablement Process [7], extra-individual factors (such as medical care and external supports) and intra-individual factors (such as lifestyle changes, coping and activity accommodations) are analogous to the extrinsic and intrinsic contextual factors discussed in the Episodic Disability Framework. While the Disablement Process classifies these factors as either interventions, exacerbators or pre-disposing factors, the Episodic Disability Framework acknowledges that all factors may exacerbate or alleviate disability depending on the specific context.

The ICF [8] also considers environmental and personal contextual factors, but while environmental factors are thoroughly described [64], personal factors are not classified because of the "large social and cultural variance associated with them" [8]. Variations in personal attributes are prominent within the HIV population with sub-groups spanning a range of cultural backgrounds and age groups. As a result, sub-classifications of individuals based on these factors are commonly reported in HIV research (e.g., men who have sex with men, people who use intravenous drugs, and individuals living with HIV from countries where HIV is endemic). Despite their complexity, their influence on the disability experience is important to consider. In response, the Episodic Disability Framework attempts to further classify these intrinsic factors as personal attributes and living strategies, and describe how differences in these factors might influence disability.

### Implications for assessing disability and future research

Our findings suggest that clinicians who are assessing disability with their clients should consider not only the dimensions of disability, but the contextual factors that may influence episodes of disability. Modifiable contextual factors may also represent possible targets for interventions to prevent or reduce disability for adults living with HIV. For example, facilitating access to supportive networks, rehabilitation programmes and services (social support), reducing stigma with education (stigma), and enhancing positive lifestyle changes (living strategies) could help reduce episodes of disability.

Future research that explores how non-modifiable personal attributes, such as aging, influence disability, may indicate age-appropriate interventions that also could be used to indicate ways to prevent or diminish disability for older adults living with HIV.

Finally, while these contextual factors were derived from the HIV-perspective, they may have broader applicability to individuals living with other lifelong illnesses who share a similar episodic disease trajectory. Further research is needed to determine similar and/or unique contextual factors to HIV and how they may or may not differ in the way they influence disability.

## Conclusion

Contextual factors within the Episodic Disability Framework offer new considerations about how disability is experienced by adults living with HIV. Extrinsic factors (level of social support and stigma) and intrinsic factors (living strategies and personal attributes) may exacerbate or alleviate episodes of HIV-related disability. These factors provide a broader understanding of the features that shape the disability experience and offer a foundation from which to build strategies to mitigate episodes of disability experienced by adults living with HIV.

## Competing interests

The authors declare that they have no competing interests.

## Authors' contributions

KO conceptualized the research question and study design, performed data collection, analysis and interpretation, and drafted the manuscript. This research was completed as part of KO's PhD thesis research study. AB and AD (co-supervisors) and CS and NY (committee members) facilitated in the conceptualization of the research question and study design, oversaw the analysis and interpretation, and revised the manuscript for important intellectual content. All authors read and approved the final manuscript.
